# Is Peripheral Immunity Regulated by Blood-Brain Barrier Permeability Changes?

**DOI:** 10.1371/journal.pone.0101477

**Published:** 2014-07-02

**Authors:** Erin Bargerstock, Vikram Puvenna, Philip Iffland, Tatiana Falcone, Mohammad Hossain, Stephen Vetter, Shumei Man, Leah Dickstein, Nicola Marchi, Chaitali Ghosh, Juliana Carvalho-Tavares, Damir Janigro

**Affiliations:** 1 Cerebrovascular Research, Cleveland Clinic Lerner College of Medicine, Cleveland, Ohio, United States of America; 2 Department of Neurosurgery, Cleveland Clinic Lerner College of Medicine, Cleveland, Ohio, United States of America; 3 Department of Biomedical Engineering, Cleveland Clinic Lerner College of Medicine, Cleveland, Ohio, United States of America; 4 Flocel, Inc. Cleveland, Ohio, United States of America; 5 Kent State University, Kent, Ohio, United States of America; 6 Department of Psychiatry, Cleveland Clinic Lerner College of Medicine, Cleveland, Ohio, United States of America; Washington University, United States of America

## Abstract

S100B is a reporter of blood-brain barrier (BBB) integrity which appears in blood when the BBB is breached. Circulating S100B derives from either extracranial sources or release into circulation by normal fluctuations in BBB integrity or pathologic BBB disruption (BBBD). Elevated S100B matches the clinical presence of indices of BBBD (gadolinium enhancement or albumin coefficient). After repeated sub-concussive episodes, serum S100B triggers an antigen-driven production of anti-S100B autoantibodies. We tested the hypothesis that the presence of S100B in extracranial tissue is due to peripheral cellular uptake of serum S100B by antigen presenting cells, which may induce the production of auto antibodies against S100B. To test this hypothesis, we used animal models of seizures, enrolled patients undergoing repeated BBBD, and collected serum samples from epileptic patients. We employed a broad array of techniques, including immunohistochemistry, RNA analysis, tracer injection and serum analysis. mRNA for S100B was segregated to barrier organs (testis, kidney and brain) but S100B protein was detected in immunocompetent cells in spleen, thymus and lymph nodes, in resident immune cells (Langerhans, satellite cells in heart muscle, etc.) and BBB endothelium. Uptake of labeled S100B by rat spleen CD4+ or CD8+ and CD86+ dendritic cells was exacerbated by pilocarpine-induced *status epilepticus* which is accompanied by BBBD. Clinical seizures were preceded by a surge of serum S100B. In patients undergoing repeated therapeutic BBBD, an autoimmune response against S100B was measured. In addition to its role in the central nervous system and its diagnostic value as a BBBD reporter, S100B may integrate blood-brain barrier disruption to the control of systemic immunity by a mechanism involving the activation of immune cells. We propose a scenario where extravasated S100B may trigger a pathologic autoimmune reaction linking systemic and CNS immune responses.

## Introduction

There is overwhelming evidence showing that systemic immunity is regulated by brain activity [1] and that this axis can be exploited therapeutically to treat CNS disease [2]. One of the chief regulators of the acquired immune response, the spleen, is under the direct or indirect influence of the central nervous system [1], [3]. This is most evident in pathophysiological models such as stroke or *status epilepticus* where immunosuppression by splenectomy exerts a beneficial effect by hampering leukocyte activation [4]–[6]. In addition to hard-wired connections linking the CNS to the immune system, soluble, circulating molecules act to modulate immunity. Adrenocorticotropic hormone (ACTH) released by the pituitary triggers a distal, cortisol-dependent immune response. There are no known protein-mediated signals which, after being released by brain cells, elicit a direct peripheral immune response of potency comparable to ACTH.

A common event in neurological diseases is increased cerebrovascular permeability [6], [7]. Whether blood-brain barrier disruption (BBBD) is a consequence or cause of the associated pathology remains unclear, but immunomodulation in seizure models protects the brain via improved BBB function. Similarly, multiple drug resistant pediatric patients benefit from treatments aimed at improving cerebrovascular integrity and decreasing systemic inflammation [8]. There is growing evidence demonstrating that seizures are in part a “BBB disease” and perhaps similar to multiple sclerosis a strong immunological component is present in epileptogenesis [6], [9]–[12].

S100B is an astrocytic protein that has been used as a peripheral reporter of blood-brain barrier disruption [8], [13]–[15]. The ratio of cerebrospinal fluid S100B compared to serum is 10∶1; this forms the bases for an ideal peripheral marker of BBBD [16]–[19]. While an unequivocal role for S100B is still lacking, evidence linking S100B to immunity is based on its interaction with RAGE receptors [20]. In addition to the brain, S100B is also present in fat tissues, skin, *etc*. [21]–[23] but results appear to be strongly dependent on the method of detection used [24]. Whether presence of S100B in peripheral organs is due to active transcription or uptake from circulating protein remains unclear. mRNA expression of S100B has been found consistently only in glia, *e.g*., astrocytes and oligodendroglia. Reports suggest that pathogen exposure or insults such as ischemia may induce S100B expression at the messenger level [25]–[27].

Degenerative brain diseases are often due to an autoimmune process. A common interpretation of the mechanism of the pathogenesis of brain autoimmunity is based on antigen unmasking by loss of CNS immunoprivilege [28], [29]. S100B autoantibodies are found in Alzheimer's and other CNS diseases [30]–[35]. The development of autoimmunity against CNS antigen may follow repeated exposure of antigen *in situ* (e.g., neuronal epitopes) or after extravasation in serum, as following BBBD. Recent findings showing anti-self IgG accumulation in epileptic human brain support this hypothesis [36].

A recent report has linked the extravasation in serum of the astrocytic protein S100B to an autoimmune response after sub-concussion-induced serum level surges [37]. These results also pointed to altered BBB function as a mechanism of long-lasting neurological sequelae. However, in spite of the wealth of literature linking S100B to the immune system [27], [38], virtually nothing is known on the systemic fate of brain-derived, trans-BBB extravasated S100B protein.

Given the fact that BBBD and subsequent S100B appearance is serum is a hallmark of many acute or chronic neurological diseases [39]–[45] as well as in animal model of seizures [13] or in human epilepsy [46], we wished to determine the fate of circulating S100B in control or post-*status epilepticus* animals. We also wished to test the hypothesis that in clinical epilepsy S100B surges precede seizures as shown in experimental models. In addition, we tested the hypothesis that S100B after accomplishing its role as reporter of BBBD also acts as a trigger of autoimmunity due to its preferential homing into immune cells.

## Methods

### Ethics statement

All experiments were performed conforming to the guidelines of the Declaration of Helsinki. All patients signed an informed consent according to institutional review protocols (IRB) at the Cleveland Clinic Foundation. Human research was conducted as per the IRB guidelines (approved protocol at Cleveland Clinic - IRB 07-322; PI-Dr Janigro). The IRB at Cleveland Clinic Foundation has specifically approved this study.

### Animal studies

Procedures involving animals and their care were conducted in conformity with the institutional guidelines that are in compliance with international laws and policies (EEC Council Directive 86/609, OJ L 358, 1, Dec.12, 1987; Guide for the Care and Use of Laboratory Animals, U.S. National Research Council, 1996). Cleveland Clinic IACUC approved the protocol number 08491 for the performance of the presented experiments. Rats were housed in a controlled environment (21±1°C; humidity 60%; lights on 08:00 AM - 8:00 PM; food and water available *ad libitum*). Rats were sacrificed by isoflurane overdose.

### Tissue analysis (rats)

All experiments were performed between 12pm and 5pm. Male Sprague-Dawley (250 gm) rats were first anesthetized with ether and sacrificed via immediate decapitation by a sharp guillotine. Peripheral blood was first removed from via intracardial puncture, after which they were perfused with saline followed by paraformaldehyde prior to decapitation. After decapitation, all necessary organs were quickly removed, placed into and rinsed with cold PBS and sliced into small sections. Tissue samples designated for sectioning and immunohistochemistry were placed into tubes containing 10% formalin for 24–48 hrs at 4°C, then transferred into 20% sucrose solution at 4°C until sunken (48–72 hrs). Tissue sections destined for RNA isolation and analysis were placed into Falcon tubes containing appropriate amounts of RNAlater solution (Qiagen); specimens were placed overnight at −4°C and then into −80°C for storage.

### S100B and S100A1 labeling

A stock solution (1 mg/ml) of the protein (bovine S100B, Cat # 559290, Calbiochem) was made by adding 1 ml of PBS to the 1 mg vial. A 1 M solution of sodium bicarbonate pH ∼9.0 then was made and stored at 4°C for 2 weeks. Then, 50 µL of 1 M bicarbonate was added to 0.5 mL of the 1 mg/mL protein solution. A vial of reactive dye was allowed to warm to room temperature (RT). The protein solution then was transferred to the vial of reactive dye (2 µL of S100B (1 µg/ml), 100 µL of AF 488, 5 µL of sodium bicarbonate and 400 µL PBS for 500 µL total) and the vial slowly inverted a few times to fully dissolve the dye; the reaction mixture was stirred for 1 hour at RT. An elution apparatus was assembled by securing the column upright, cushioned by a provided foam holder, to a ring stand with a clamp; a funnel was attached to the top of the column and the bottom cap removed once a beaker had been placed underneath.

Elution buffer was prepared by diluting the room temperature 10X stock (0.1 M potassium phosphate, 1.5 M NaCl, pH 7.2, with 2 mM sodium azide) 10-fold in dH2O and warmed to RT. Bio-Rad BioGel P-6 was used as a purification resin and added into the column until resin was ∼3 cm from top of column, with excess buffer being allowed to drain into the beaker. Elution buffer was added to the column prior to application of the protein mixture, ensuring a consistent and even flow; column was repacked if flow was unsatisfactory. The reaction mixture was loaded directly onto the resin column via pipette. The reaction vial was rinsed with ∼100 µL of elution buffer and this was applied to the column. Elution buffer was added slowly, gathering 1.5 mL fractions once the labeled protein began eluting, until all of it was eluted (confirmed by use of a UV light to view fluorophore in the column). This same procedure was utilized to create AlexaFlour 594-tagged S100A1 (Human Recombinant protein, Genway Id# GWB-D23339); in this case, protein concentration was 10 µg/78 µl and a working stock of 1 µg/1 mL was made by adding 7.7 µL of original stock +992.3 ml PBS. 2 µl from this solution was used to make the reaction mixture. Tagged protein was stored in foil-wrapped packages at -20°F.

### Injection

Rats were anesthetized with inhaled isofluorane via mask during the procedure. Isolated S100B protein tagged with AlexaFlour 488 fluorophore at a 1∶10 ratio with saline (15 µL protein in 135 µL saline) was injected into the tail vein and the animal allowed to recover for 3–4 hrs prior to sacrifice and harvesting of tissues.

### Immunohistochemistry

For immunohistochemistry, tissue sections mounted on slides were first washed x 3 with PBS, then incubated 1–2 hrs in blocking solution made of 3% NGS, Triton X and BSA in TBS. Slides were then incubated in primary antibody in blocking solution (approximately 0.5 mL/slide) overnight at 4°C. Afterwards, slides were washed x 5 with PBS, incubated in secondary antibody in blocking solution (approximately 0.5 mL/slide) for 2 hrs in the dark and kept in the dark thereafter. Slides were washed x 3 in PBS, then for single immunolabelling, mounting was performed by application of one drop Vectashield mounting medium with DAPI onto slide followed by cover slip; slides were stored at −20°C. Mouse anti-S100B (Sigma) as primary antibody at 1∶100 dilution and donkey anti-mouse conjugated to FITC (Jackson) at 1∶200 dilution as secondary antibody were used for single labeling of S100B. For double immunolabelling, initial labeling of S100B was accomplished using rabbit anti-S100B (Chemicon) primary and donkey anti-rabbit conjugated to Texas Red (Jackson) at a 1∶200 dilution in order to be compatible with the necessary secondary for CD4/CD8 labeling. Second labeling was accomplished using mouse anti-rat CD4 or CD8 (BD Pharmingen) at 1∶40 dilution as primary and donkey anti-mouse conjugated to FITC (Jackson) at 1∶200 dilution. The dendritic cell marker CD86 (1∶100 Abcam; rabbit monoclonal) and the endothelial cell marker CD31 (1∶50 Abcam; rabbit polyclonal) were used as primary antibodies. Donkey anti-rabbit conjugated with Texas Red (Jackson) at a dilution of 1∶100 was used as secondary antibody.

### RNA isolation and analysis

RNA was isolated via RNeasy Mini Spin Kit (Qiagen); tissue specimens frozen at −80°C in RNAlater solution (Qiagen) were allowed to thaw before 50–100 mg were removed and homogenized in QIAzol Lysis Reagent with a pestle and glass tube. Tissue homogenate was then processed according to RNeasy Mini Spin Kit protocol (Qiagen); a series of buffers and centrifugations were used to wash the RNA bound to the membrane of the column, with 50 µL RNase free water being used to elute the bound RNA. RNA extracts were tested via spectrophotometry for purity and concentration, using a cutoff value of 1.6 (Abs 280 nm/Abs 260 nm) to determine acceptable RNA purity.

### RT-PCR

The Superscript III One-Step RT-PCR with Platinum Taq kit (Invitrogen) was used to prepare all samples. Each sample of RNA was prepared using 3 µg respective RNA isolate, 25 µL 2x Reaction Mix, 1 µL each of forward and reverse primers (10 µM), 2 µL Superscript III RT/Platinum *Taq* Mix and autoclaved distilled H_2_O to a final volume of 50 µL. Two negative controls were prepared using all reagents except for RNA or enzymes. For S100B primer sequences used were (5′-3′) F: TTGCCCTCATTGATGTCTTCCA, R: TCTGCCTTGATTCTTACAGGTGAC. All samples were placed into an automated thermocycler with an initial cycle of 30 min at 55°C, followed by 2 min and then 20 sec cycles at 95°C, a 30 sec cycle at 50°C and then 90 sec cycle at 70°C. The cycles from 2 min at 95°C onward were repeated either 35 or 40 times, before a final cycle of 5 min at 70°C and cooling thereafter.

### Pilocarpine status epilepticus

Rats (male *Sprague-Dawley* 225–250 g) were injected with methylscopolamine (0.5 mg/kg, i.p., Sigma-Aldrich) and 30 minutes after with pilocarpine (340 mg/Kg, Sigma-Aldrich). We have analyzed data obtained from a total of 6 rats. Development of seizure and status epilepticus were evaluated by behavioral (Racine's scale) and EEG assessment. Animals that did not develop *status epilepticus* were not used for the experiments shown herein (n = 1, not counted in the 6 total). The pilocarpine model of seizures has been characterized by many laboratories and has become the standard method for induction of temporal lobe epilepsy in rodents [47]. For the purpose of our study we have only focused on the acute *status epilepticus* model induced by pilocarpine e.g., [8], [9], [48] but animals which develop acute seizures will spontaneously become epileptic after a lag time of weeks [47].

### BBBD in human subjects

All patients signed an informed consent according to institutional review protocols at The Cleveland Clinic Foundation and the Declaration of Helsinki. Human research was conducted as per Institutional Review Board (IRB) guidelines (approved protocol at Cleveland Clinic - IRB 07-322). Newly diagnosed immunocompetent patients with Primary CNS Lymphoma (PCNSL; n = 3) were enrolled in the study and prospectively treated by the BBBD program at the Cleveland Clinic Foundation. This protocol involves concurrent intravenous chemotherapy and a treatment protocol including BBBD [49] followed by intra-arterial instillation of chemotherapy (IAC). Eligible patients had histologically confirmed PCNSL (by brain biopsy or CSF cytology), with no evidence of lymphoma elsewhere in the body and no HIV infection at diagnosis. Before treatment, patients were required to have an absolute granulocyte count more than 1,200/µL, platelet count more than 100,000/µL, and normal hepatic and renal function. Patients with uncontrolled pulmonary or cardiac complications were not eligible. During this multicenter study, three patients with histologically proven non-acquired immunodeficiency syndrome Primary Central Nervous System lymphoma (PCNSL) consented to participate in an IRB-approved protocol for the management of this disease at the Cleveland Clinic Foundation; this subset of patients also agreed to additional blood draws at time of iatrogenic BBBD procedures. The details are summarized in [50].

Specifically, three patients were treated with intra-arterial injection of mannitol causing a temporary disruption of the BBB followed by a selective intracarotid chemotherapic injection [13], [49], [50]. The procedure consists of the following steps: 1) Patient is taken to the operating room and general thiopental anesthesia is induced. 2) Catheterization of a selected intracranial artery (either an internal carotid or vertebral artery) is performed via a percutaneous transfemoral puncture on a given treatment day. 3) Mannitol (25%) is administered intra-arterially via the catheter at a predetermined rate of 3–12 cc/sec for 30 seconds. 4) After the BBB is opened with mannitol, intra-arterial Methotrexate is infused. No seizures were associated with injection of contrast or chemotherapy in the absence of mannitol. 5) Immediately following delivery of chemotherapy, non-ionic contrast dye is given intravenously. 6) The patient is transported, still anesthetized, for a computed tomography (CT) scan. This step is essential to determine and document the degree of BBB opening since better disruption portends better chemotherapy delivery across the barrier. Methods for grading the degree of BBBD and correlation of these grades with Hounsfield units were previously described [51]; degree of BBBD was graded by visual inspection as nil, fair, good, or excellent. 7) After the CT scan is completed the patient is awakened, extubated and monitored in the hospital overnight. This is a 2 day procedure whereby two different intracranial vessels (typically an anterior circulation vessel, left or right internal carotid artery, on one day and the contralateral posterior circulation vessel right or left vertebral artery on the following day) are cannulated on consecutive treatment days for BBBD and instillation of chemotherapy.

### Ictal S100B measurements

Patients were consented in the monitoring unit and after a brief explanation of the study a dedicated investigator was assigned to the patient. The consent form used for this purpose was approved by the IRB committee and was part of a broader study on the diagnostic use of serum markers for neurological disorders. A blood sample was drawn after consent was obtained (pre-ictal or interictal). We noted the time interval from previous seizure; only >3 hrs. from seizure samples were used for this purpose. We then draw another 1cc sample at time of scalp EEG changes consistent with seizure onset. When a false positive sample was taken, i.e., no seizure followed EEG changes, the sample was discarded. All patients suffered from multiple drug resistant seizures and were evaluate for brain resections.

### Serum S100B and auto-antibody measurement

We used colorimetric immunosorbent assay, Sangtec 100 ELISA, by DiaSorin, Inc. (Stillwater, MN) to quantify S100B. The limit of detection is 0.03 ng/mL. The process used is analogous to previous published work [24], [43], [52]–[56].

Auto-antibodies against S100B were measured in serum samples via ELISA. Maxisorp ELISA 96-well plates were coated with a PBS solution containing S100B protein (Human brain, catalog number-559291, EMD chemicals). See reference [37].

## Statistical Analysis

Origin 8.0 (Origin Lab, Northampton, MA, USA) and Jump 9.0 software were used. For all parametric variables, differences between populations were analyzed by ANOVA. In all figures, symbols with error bars indicate mean ± SEM; *p<0.05 was considered statistically significant and *p* values are shown in the figures or accompanying legends.

## Results and Discussion

For design of the rodent experiments presented herein we used a total of 16 rats; 5 animals were used for protein uptake experiments, 5 were used for immunohistochemistry. An additional 6 animals were used for the pilocarpine experiments. Of the 5 animals used for protein uptake, one also was injected with an equal part mixture of S100B and S100A1; the remaining animals were used for experiments of distribution of labeled S100B. In addition, serial samplings from 3 patients during a long-term BBBD treatment in patients with PCNSL were used for human data. Blood samples from 11 epileptic patients affected by multiple drug resistant seizures were used to measure ictally or interictally serum levels of S100B.

### S100B mRNA expression is limited to barrier organs

**Figure 1:**
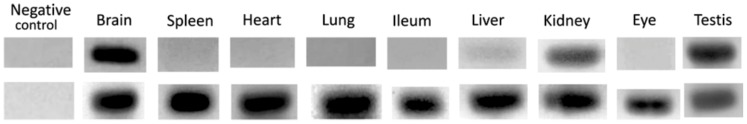
S100B transcription only occurs in barrier organs. Reverse-transcriptase PCR results for S100B (*top row*) and β-actin (*bottom row*) mRNA in various rat tissues. S100B mRNA was found in brain tissue and testis; kidney tubule cells also expressed measurable levels of S100B mRNA. No S100B mRNA was found in organs where S100B protein levels were previously demonstrated by us and others (see [24]). These results summarize outcomes from at least four repetitions. Changing the number of PCR cycles in the protocol from 40 to 35 (see Methods) did not result in any significant changes.

Messenger RNA (mRNA) analysis was performed on tissue samples isolated from the same animals used for immunohistochemistry experiments. In a previous study [24], we demonstrated S100B protein content by Western blot analysis in human or rat organs. Figure 1 shows the pattern of mRNA expression in various organs (see also Figure S1). Only brain, kidney and testis demonstrated measurable S100B mRNA levels; all other organs tested displayed no measurable mRNA levels. A summary of these findings is also shown in Table 1. The presence of mRNA was thus restricted to barrier organs; in fact, kidney cells contributing to the mRNA signal were post-glomerular tight junction-forming tubular cells (*data not shown*).

**Table 1:** Summary of experimental results testing for the presence of S100B at the protein, mRNA levels or after injection of labeled S100B.

	Cellular uptake of labeled S100B	Immunohistochemistry	mRNA	β actin
**Brain**	-	++	+	+
**Heart**	+	-	-	+
**Lung**	+	-	-	+
**Liver**	+	-	-	+
**Kidney**	+	-	+	+
**Spleen**	++	++	-	+
**PBMC**	+	+	-	+
**Testis**	-	++	+	+
**Eye**	-	-	-	+
**Ileum**	+	-	-	+
**Skin**	-	-	-	+
**Lymph node**	++	+	ND	+
**Thymus**	++	+	-	+

+ indicates presence, ++ presence at levels significantly greater than in tissues labeled with +. ND =  Not determined, - indicates absence of measurable signal.

### Circulating S100B distribution in endothelial cells

**Figure 2:**
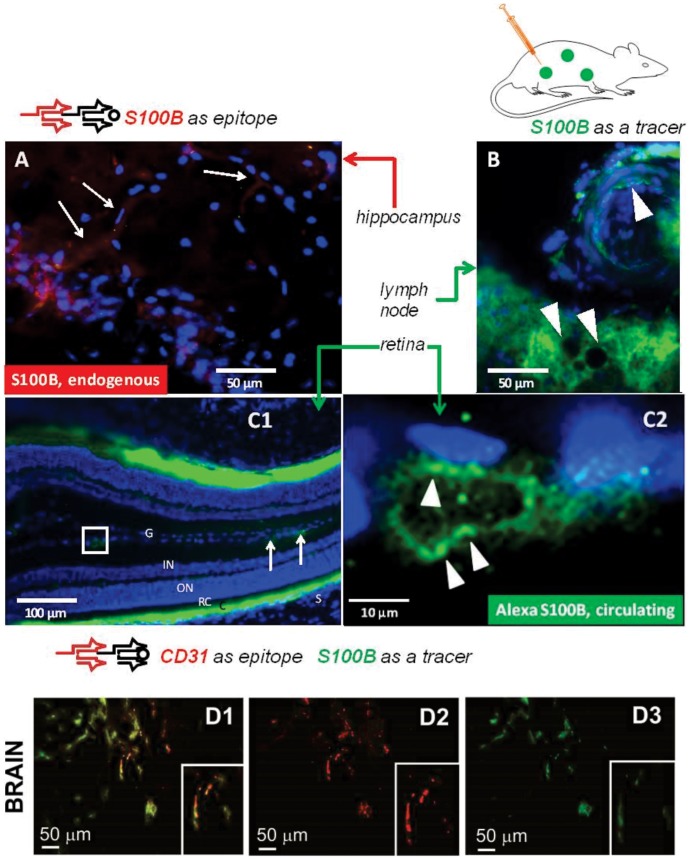
Endothelial cells take up circulating but not CNS-derived S100B. (**A**) Lack of significant endogenous immunoreactivity for S100B in BBB endothelial cells. The *arrows* point to faintly stained capillaries in the hippocampus (CA1 region). This staining was accounted for by glial end feet positive for S100B. Note the endogenous S100B immunostaining of astrocytes located within the pyramidal cell layer. (**B**) Uptake of circulating (exogenous) S100B by endothelial cells after injection of green S100B is observed in most systemic vessels. The example shows the appearance of an arteriole and capillaries in a lymph node, indicated by *arrowheads*. (**C1**) and (**C2**) show the uptake of exogenous, circulating S100B by endothelial cells of the retinal barrier. Note the uptake indicated by *arrows* and the various retinal layers. (**D1-3**) Co-localization of CD31 and exogenous S100B indicates uptake by endothelial cells. Endothelial cells take up S100B after systemic injection of labeled protein. The results shown here use an immunohistochemical validation by an endothelium-specific marker to corroborate the results in Figure 1. In fact, the cells demonstrating S100B uptake (*green*) were also positive for the endothelial marker CD31. See also Figure S3 A for CNS BBB endothelial cells. *G =  ganglion cell layer, IN  =  inner nuclear layer, ON  =  outer nuclear layer, RC  =  rods and cones, C =  choroid, S =  sclera*. AF 488-tagged S100B was injected to achieve a serum concentration of 0.12 ng/mL to mimic blood-brain barrier disruption (ref. 13).

Specialized endothelial cells of the blood-brain barrier segregate brain S100B from the systemic circulation. Under physiologic conditions, S100B is present in the brain extracellular fluid, CSF and glia. Since astrocytes, the chief expressors of S100B, extend their endfeet to contact the basal lamina juxtaposed to BBB endothelial cells, it is possible that S100B will remain entrapped in cells lining the vascular wall. We thus tested whether capillary endothelial cells are capable of S100B uptake. The results in Figure 2A show that in spite of the close proximity between endothelial cells of the BBB and glia, endogenous S100B (in *red*) is not present, or is present only at low levels in endothelial cells. In contrast, circulating, exogenous S100B (in *green*) was readily taken up by endothelial cells involved in barrier function. The data shown in Figures 2C1 and C2 refer to the retinal barrier endothelial cells but similar results were observed in other CNS regions as shown in Figure 2D1-3. Note that the exogenous S100B protein (in green) colocalized with the endothelial cell marker CD31 (in red). These results suggest that an asymmetric uptake of this CNS protein may exist at the endothelial cells in the CNS. Thus, S100B from the lumenal but not ablumenal side may undergo endothelial cell uptake. This uptake process was not specific for brain endothelial cells since in other organs (*e.g*., lymph nodes, Figure 2B) endothelial cells also displayed pronounced uptake of exogenous, Alexa-labeled S100B.

### S100B expression and uptake in barrier organs

**Figure 3:**
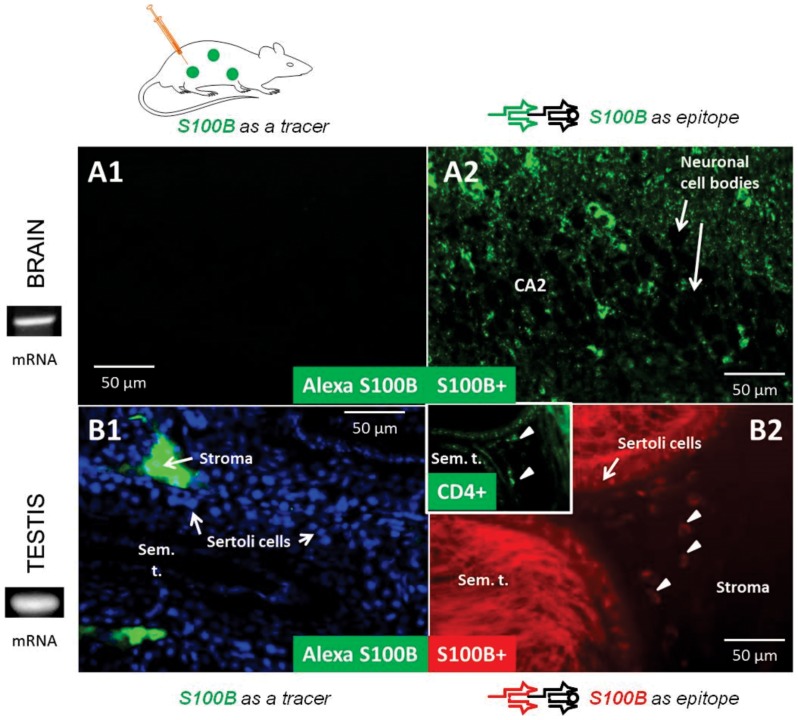
Circulating S100B fails to invade barrier organs; however S100B gene transcription and protein synthesis occurs in both brain and testis. Injection (exogenous) strategies demonstrate privilege of barrier organs to transendothelial diffusion of S100B while immunodetection of endogenous S100B demonstrates brain- and testis specific S100B protein by astrocytes and Sertoli cells. mRNA detection in the same barrier organs confirms S100B expression. **(A1)** shows the lack of fluorescent signal (Alexa Fluor (AF) 488, in *green*) in brain regions where endogenous S100B was readily detected **(A2)**. The section used for immunohistochemistry contained the CA2 sector of the hippocampus. Note that, as expected, S100B was present in glial cells but not in neurons; neuronal cell bodies in hippocampal CA2 region are seen as unstained ghosts. Testicular tissue yielded similar results albeit in testis the barrier is established by Sertoli and not endothelial cells **(B1 and B2)**. Note that intravascular S100B was restricted to the stroma of the seminiferous tubules in the testis where endogenous S100B was not present. S100B+ cells in the stroma (*arrowheads*) are CD4+ dendritic cells (*insert* in **B2**). DAPI (*blue*) was added as a nuclear stain **(B1)**. AlexaFlour 488-tagged S100B was injected to mimic blood-brain barrier disruption (e.g., ref. 13). The labeled protein was allowed to circulate 3 hrs. prior to tissue harvesting. The mRNA bands shown reflect levels of expression by brain and testis. *Sem. T.  =  seminiferous tubule*.

Intravenous injection of labeled S100B was performed to achieve steady-state blood levels comparable to those observed after leakage of S100B across the blood-brain barrier. These levels vary in the range from 0.01 ng/ml to 0.1 ng/ml [15], [18], [19] and also recapitulate S100B levels measured after *status epilepticus* [8], [9], [13]. S100B is primarily expressed by brain glia [57]. We initially focused on the analysis of protein levels after intravascular injection in barrier organs. The results are shown in Figure 3. As expected, given the fact that S100B is impermeant across an intact BBB, no exogenous (labeled with AF488) S100B was detected in brain (Figure 3A1). Identical patterns were determined in testicular samples (Figure 3B1) where the only exogenous S100B was found in the stroma or in the intravascular compartment where S100B was injected.

An almost symmetrical set of results was obtained when analyzing brain and testis to measure endogenous S100B by immunohistochemistry (Figures 3 A2 and B2). Given the predominant expression of S100B by brain astrocytes, widespread staining was observed in cortical and hippocampal sections. An example is shown in Figure 3B where the CA2 region of the hippocampus is shown. As expected, neuronal cell bodies were negative for S100B immunoreactivity owing to the glia-specific expression pattern of this protein. Testicular sections stained for S100B by immunohistochemistry revealed robust staining of Sertoli cells – involved in sperm cell generation and development and maintenance of the blood-testicular barrier – as well as cells containing spermatocytes at various levels of maturation. In addition, sparse staining limited to a few cells in the stromal region revealed that these consisted of CD4+ dendritic cells [58]. This was also confirmed by CD4+ and CD8+ (*not shown*), as well as by their location in the stroma and absence from the circulatory system.

The results in Figure S2 show that use of a 1∶1 (S100B to vehicle) solution results in intense staining, while use of a 1∶10 solution produced staining comparable to what was observed by immunohistochemistry. This suggests that, in most peripheral organs, S100B levels at steady state are too low to activate cellular uptake in non-immune cells. Increased serum S100B, as seen after BBBD, leads however to greater uptake even in cells with no known immune function.

The result so far presented show that endogenous expression of S100B at the mRNA and protein level is confined to organs characterized by the presence of a barrier - namely kidney, testis and brain - while the distribution of protein also encompasses a distinct signal in cells immune positive for the dendritic cell or lymphocytic marker (*inset* in Figure 3B2).

### Serum S100B distributes into immune cells

**Figure 4:**
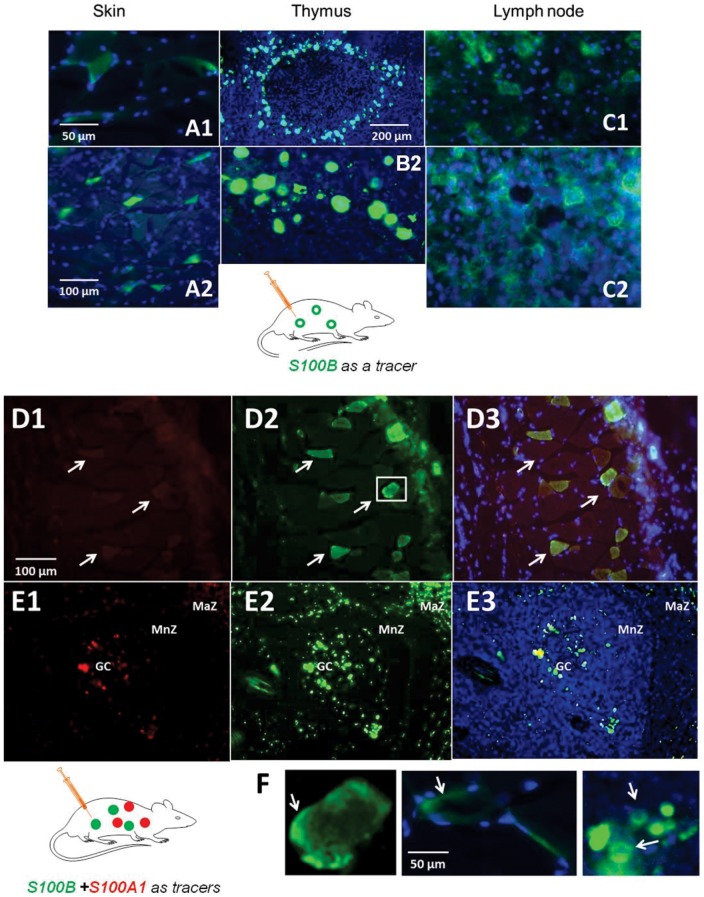
Circulating S100B is taken up by peripheral immune cells. AlexaFlour 488-tagged S100B protein accumulated in Langerhans cells of the skin (**A1, 2**), thymic dendritic cells (**B1, 2**), and in dendritic cells in lymph nodes (a para-aortic lymph node is shown in **C1, 2**). Note the lack of uptake by non-immune surrounding tissue. Also note the typical appearance of dendritic cells in nodal tissues. Injection consisted of AlexaFlour 488-tagged S100B at a concentration of 0.12 ng/mL introduced via tail vein and allowed to circulate 2–3 hrs. prior to tissue harvesting; DAPI (*blue*) was added as a nuclear stain. Note the different magnifications among the panels, with scale bar  = 200 µm in B1, 100 µm in A2 and 50 µm in all other panels. Rats were injected with a combination of AlexaFlour 594-tagged S100A1 (*red*) and AlexaFlour 488-tagged S100B (*green*). Skin (**D**) and splenic (**E**) tissue revealed that Langerhans cells (*arrows* in D) and splenic cells within the germinal center (*GC*), mantel zone (*MnZ*) and marginal zone (*MaZ*) demonstrate uptake of both S100A1 and S100B; however, the extent and intensity of S100B uptake was much greater as also evident in the merged figures (D3, E3). **F** shows the preferential segregation of S100B at the membrane of dendritic cells in skin (**F1** and **F2**) and thymus (**F3**); greater detail of a Langerhans cell from D2 (box) is shown in F1 in order to highlight membrane staining. Injection consisted of AlexaFlour 488-tagged S100B at a concentration of 0.10–0.12 ng/mL introduced via tail vein and allowed to circulate 2–3 hrs. prior to tissue harvesting; DAPI (blue) was added as a nuclear stain. *GC  =  germinal center, MnZ  =  mantle zone, MaZ  =  marginal zone*.

In the next set of experiments, we examined the distribution of the exogenous and the expression of the endogenous S100B in immune cells. Figure 4 shows the results of these experiments. S100B injected intravenously accumulated preferentially in dendritic cells of the skin, known as Langerhans cells (Figure 4A), in dendritic cells of the thymus (4B) and in lymph nodes (4C). Note the fine structure of dendritic cells in a lymph node with evident membrane segregation of S100B in clusters of these cells. The dendritic cell-specific marker CD86 [59], [60] was used to demonstrate that skin cells which were responsible for the uptake of S100B were immunocompetent cells. These results are shown in Figure S3.

### Specificity of S100B uptake in immune cells

The results so far presented are consistent with an uptake of circulating S100B by dendritic cells. These cells are specialized in presentation of antigen and the uptake of protein or protein fragments; thus uptake of S100B by these cells is *per se* not surprising. S100B is an endogenous molecule that may be recognized as foreign by dendritic cells simply because of its customary absence from the systemic circulation where self-antigen presentation occurs. Whether uptake of S100B by dendritic cells was due to a process specific for this protein which is normally segregated in brain, or rather constituted the normative process for any low molecular weight protein regardless of its location, was tested in the subsequent set of experiments.

Animals were injected with the same amount of AF 488-tagged S100B as in the previous experiments. In addition, animals also received an equal dose of AF594-tagged S100A1 injected simultaneously. Both dyes bind to primary amines and have properties that allow tracking protein in tissue or fluids. The differences between the sequences of S100A1 and S100B protein are shown in Table 2. Note that both S100B and S100A1 diffused into skin or spleen dendritic cells (see Figure 4D-F). Cellular uptake of these proteins varied, but intracellular accumulation of S100B was more robust than that of its counterpart S100A1. Given the fact that both proteins were injected at the same concentrations and simultaneously, these results suggested that both S100B and S100A1 accumulate within the dendritic cells, but that uptake of S100B predominates.

**Table 2:** Comparison of S100B and S100A1 sequences to underscore similarities (normal character) and differences (in bold).

	Protein sequence for tagged tracers	Mol. Wt (kD)
S100A1 Human rec	mgseletametlinvfhahsgkegdkyklskkelkellqtelsgfldaqkdvdavdkmkeldengdgevdfqeyvvlvaaltvacnnffwens	10.4
S100B bovine	mselekamvalidvfhqysgregdkhklkkselkelinnelshfleeikeqevvdkvmetdtndgdgecdfqefmafvamvtacheffe	10.61

There is 94% (4 aa are different) similarity between bovine S100B used for these experiments and rat S100B. Thus, the uptake by immune cells is not due to foreign antigen sequence. Same considerations can be made for human S100A1 used as a tracer, since the human protein used shared 96% identity with rat protein.

In an attempt to quantify the cellular uptake of S100B or S100A1, we used the approach shown in Figure S4 where time-dependent uptake of S100B and S100A1 by dendritic cells were quantified by Q-Capture software. The cells were first extensively characterized for expression of CD86, CD11a and CD83, markers of dendritic cells. The robust uptake, albeit in less than 20% of cells was seen for S100B and not S100A1. These results are essentially the same as those reported in vivo, but cultured cells were easier to study since they constitute an ideal tool for time-dependent analysis.

### Effects of blood-brain barrier disruption on S100B distribution: experimental model of seizures

**Figure 5:**
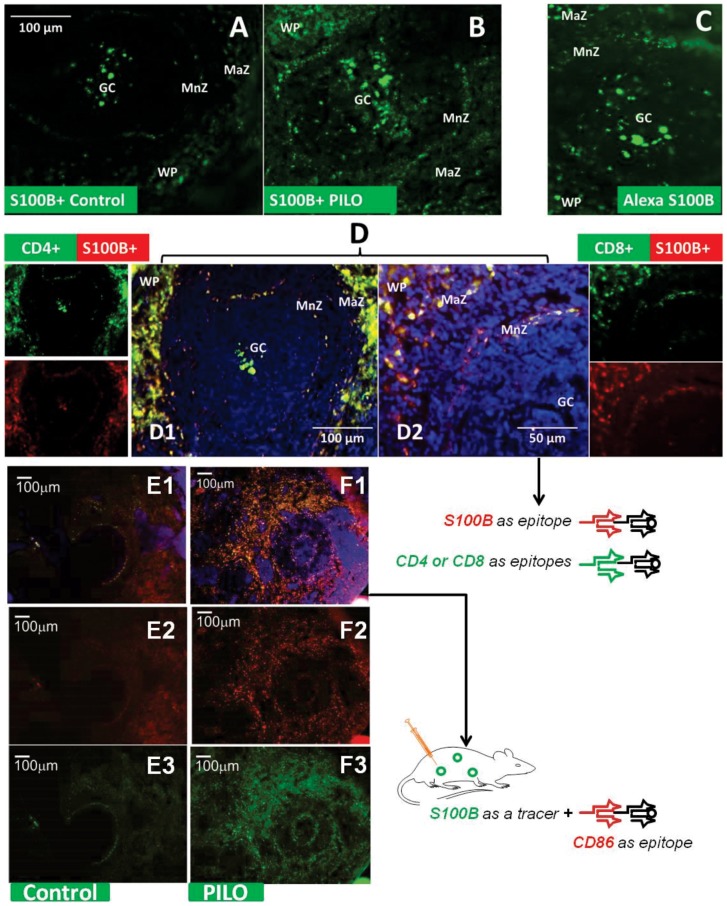
Splenic S100B-positive cells change in both number and morphology following pilocarpine-induced seizure. S100B positive cells can be viewed within splenic follicles both in an unmanipulated animal (**A**), as well as following simulation of BBBD via IV injection of Alexa Fluor 488-labeled S100B (**B**) and BBBD from pilocarpine administration (**C**). The pattern of staining is preserved in all 3 conditions, however, staining is clearly augmented in BBBD simulation and to a much greater degree in actual induced BBBD. S100B-labeled lymphocytes and dendritic cells can be observed in all regions of the splenic follicle. Note that the morphology of labeled cells also appears to change with induction of BBBD in (**C**), where cells appeared to have a more dendritic and interconnected staining pattern than in other conditions. The identity of these cells was verified via the immune cell markers, CD4 and CD8 immunostaining (**D1–D2**); S100B+ and CD4+/CD8+ double positive cells are again found throughout the follicle, with emphasized staining in the marginal zone vs other areas. Injection consisted of AlexaFlour 488-tagged S100B at a concentration of 0.10–0.12 ng/mL introduced via tail vein and allowed to circulate 2–3 hrs prior to tissue harvesting. For induced BBBD, the animal was treated with pilocarpine and spleen was removed prior to onset of status epilepticus. Sections in **A** and **C** were treated with mouse anti-S100B antibody (Ab) and donkey anti-mouse 2° Ab conjugated to FITC (Jackson). In **D1–D2**, sections were treated with rabbit anti-S100B Ab and donkey rabbit 2° Ab conjugated to Texas Red (Jackson) and rat anti-CD4 or CD8 antibody and mouse anti-rat 2° Ab conjugated to FITC (Jackson). DAPI was added as a nuclear stain. In **E–F**, sections shows rats injected with S100B tracer in control (**E1–E3**) and pilocarpine administrated rats (**F1–F3**). Co-localization of S100B+CD86 (F1) and high individual staining of CD86+ (**F2**) in pilocarpine compared to controls **E1** and **E2** indicates that activated immune cells capture S100B. The dendritic cell nature of these cells was further demonstrated by their CD86+ staining (Figure S3). Scale bar in A = 100 µm for all images. *GC  =  germinal center, MnZ  =  mantle zone, MaZ  =  marginal zone, WP  =  white pulp, RP  =  red pulp*.

One of the limitations of this study so far presented is the fact that exogenous labeled S100B was artificially injected into the blood stream in an attempt to mimic what happens under pathophysiological conditions, *e.g*., at time of seizures or after acute disruption of the BBB [8], [9], [13], [14], [61]–[63]. To compare the distribution of S100B under pathophysiological conditions, we examined the distribution of brain S100B after its crossing of the BBB in an acute model of seizures. Pilocarpine-induced seizures have been shown to depend on disruption of the BBB [8], [64]; thus, even before the onset of pilocarpine-induced *status epilepticus* S100B is elevated and the BBB breached to Evans Blue or FITC-labeled albumin [8], [9], [48], [65]. We therefore examined the intracellular content of S100B detected by immunohistochemistry in the spleen of naïve animals (Figure 5A) or in animals at time of *status epilepticus* which corresponds to a peak of S100B serum concentration (Figure 5B). After distribution of S100B into the blood stream across a leaky BBB, a pronounced increase of S100B levels in dendritic cells and lymphocytes in the spleen was observed. The findings support the hypothesis that the fates of artificially injected S100B levels are indeed comparable to the cellular distribution of S100B after blood-brain barrier disruption.

To test the hypothesis that an accumulation of S100B in cells of the immune system followed increased BBB permeability, splenic tissue was further processed for the generic lymphocytic marker CD3. We previously demonstrated that seizures can be prevented by splenectomy and that the CD3+ population of splenic cells is increased before *status epilepticus* ensues [5]. In the current study, we show that CD4+ and CD8+ cells, which are altered by seizures [5], also contain S100B (Figure 5D). We further characterized these cell populations as dendritic cells of the spleen by use of the dendritic cell marker CD86 (see Figure S3B). The marker presence co-localized with uptake of green fluorescent S100B.

### Blood-brain barrier permeability in clinical seizures

**Figure 6:**
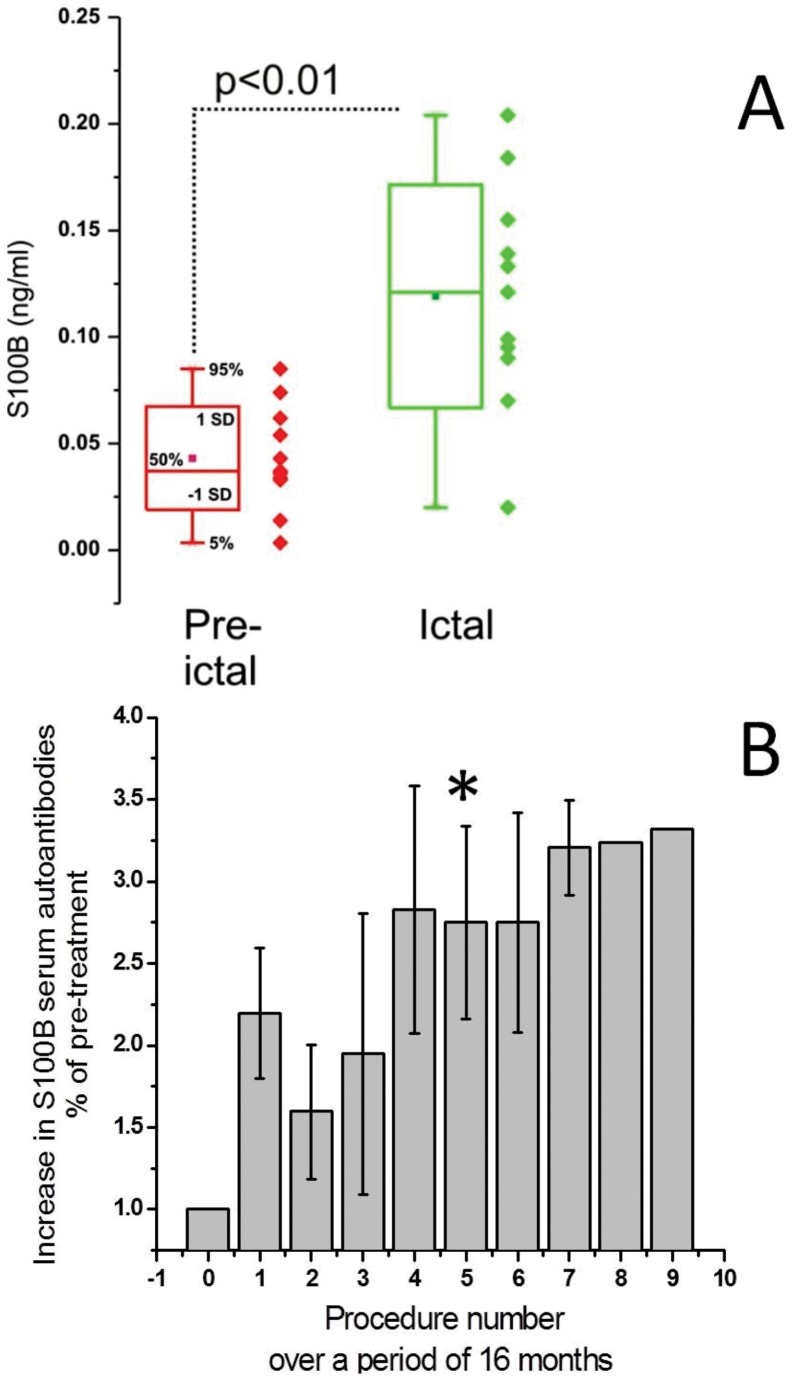
BBBD precedes clinical seizures and result in the formation of S100B autoantibodies. (**A**) Data were obtained by performing serum analysis in patients affected by seizures who underwent monitoring in the Epilepsy unit. Blood was drawn as soon as changes consistent with seizure development were observed. These consisted of a combination of behavioral and EEG changes. False positive samples (blood drawn, no subsequent seizure within 15 minutes) were discarded. Pre-ictal samples refer to blood drawn before seizures (at least 4 hours from preceding episode). Note the sharp increase in S100B at time of ictal event. The asterisks indicate p<0.05 (ANOVA). (**B**). Serum samples were taken from patients undergoing osmotic disruption of the BBB [49], [50] to improve chemotherapic efficiency. This procedure causes repeated BBBD and S100B elevations at each cycle of treatment [85]. The positive linear relationship between number of treatments with iatrogenic disruption of the BBB and % increase in serum S100B autoantibodies is shown. Auto-antibody titers show a sharp increase around treatment/month 4; after this time levels remained constant. The asterisks indicate p<0.05 (ANOVA). Statistical significance was achieved only after 5 cycles of BBBD. The data remained statistically different from pre-BBBD throughout procedure 7. These data represent the average of values for three patients undergoing treatment for primary CNS lymphoma, with induced osmotic breaching of the BBB; monthly treatments were performed for a total of 9 months. All three patients completed 6 treatments; two of the three completed 7 and one completed 9.

Experimental seizures models have shown that BBBD is a common feature of epilepsy. This was however hard to replicate in patients owing to the lack of suitable markers that can be used non-invasively. With the advent of S100B and other peripheral markers of the BBB, we were able to monitor changes in permeability in a monitoring unit setting where patients with multiple drug resistant seizures were observed by scalp EEG. Preliminary results were published elsewhere [46] but the summary of the results is shown in Figure 6A. Data from 11 patients (all adults) were averaged to show that S100B levels collected interictally (pre-ictal in Figure 6A) or immediately at time of seizure onset, as determined by the investigators who were constantly monitoring patients' EEG. When a false positive response was seen (i.e., blood was drawn but no obvious seizure was recorded) the sample was discarded. The data show a statistically significant difference between interictal/pre-ictal samples and those collected at seizure onset. These results are consistent with results obtained in experimental models [13], [48].

### Does S100B trigger an autoimmune response?

**Figure 7:**
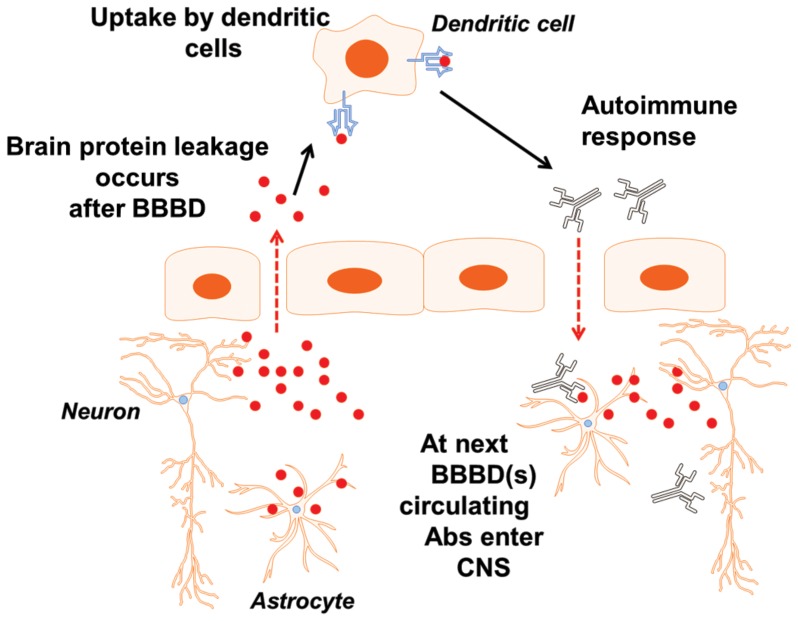
Schematic representation of the events that follow BBBD. See text for details.

A prediction of the hypothesis that S100B is recognized as a foreign antigen by dendritic cells is that an autoimmune response may results from S100B uptake by immune cells as depicted in the schematic representation in Figure 7 with BBBD. This was tested in a population of patients undergoing intra-arterial chemotherapy (Figure 6B). In a preliminary set of experiments, we demonstrated that repeated exposure of peripheral immune cells to circulating S100B results in production of autoantibodies against the same protein. The results are shown in Figure 6B, where blood samples were analyzed in a group of patients undergoing intra-arterial osmotic disruption of the BBB by mannitol as described in [13], [49], [50]. After “opening” of the blood-brain barrier by hyperosmotic mannitol administration, seizures are not uncommon [50], and these are a direct consequence of BBBD as measured by serum S100B [13], [66]. Regardless of the underlying reason for serum S100B spikes, these triggered an autoimmune response in the 3 patients analyzed. This response developed over time and approached steady state after 4 blood-brain barrier disruption procedures over a period of approximately 3 months. Whether this autoimmune response was due to leakage of the BBB, to seizures, or to a combination of these remains unknown. In addition, whether autoimmune IgGs may under conditions of BBBD enter the CNS was also unproven.

The main finding of this study is that S100B, a protein normally shielded from the systemic circulation by the blood-brain barrier can act as an immunoregulator after disruption of the endothelial tight junctions that separate the brain from the blood. An additional significant finding from our work is that, upon release in the systemic circulation, the astrocytic protein S100B is taken up by immune cells throughout the body. A corollary finding that needs to be further supported by larger scale studies is that the activation of immune cells by circulating S100B may result in an autoimmune response. This response was seen in patients affected by brain neoplasms, but experimental results show that this autoimmune potential can be reproduced in rodent models of acute seizures. In addition, we have confirmed preliminary results showing that the BBBD reporter S100B is elevated at time of onset of clinical seizures, suggesting that the systemic immunomodulation by extravasated S100B may be a mechanism of chronic epileptogenesis.

S100B has been used for many years as a reporter of blood-brain barrier dysfunction [19], [55] or in other studies as a marker of brain damage [67], [68]. Once secreted/released, S100B exerts autocrine and paracrine effects on responsive cells by engaging the receptor for advanced glycation end products [69]. Beyond the significance of S100B as a RAGE receptor activator, our results have shown that this molecule may act as a diffusible signal that connects the function of the brain to the immune system. In contrast to what previously thought, S100B may specifically connect BBB disruption to innate or acquired immune response. Downstream influences mediated by the vagus nerve influence immunity and hormones such as cortisol can have profound effect on immunity [70], [71]. To our knowledge this is the first report linking a brain-derived protein to an immune response in the context of normal immunological function. In fact, the animals used for our experiments were normal (with the exception of the seizure model used), and therefore the results can be interpreted as physiological occurrences under conditions of normal BBB function. In naïve animals S100B is highly expressed by glia and absent in neurons. Detectable levels of S100B protein (but not mRNA) were measured in cells of the immune system such as lymph nodes, spleen, skin, heart muscle, dendritic cells, *etc*. In contrast, barrier organs such as testis and kidney expressed both S100B protein and mRNA. It therefore appears that S100B can be both produced locally (in barrier organs), or taken up by cells in various systemic tissues. In addition to immune cells, we also measured uptake of S100B by brain endothelial cells, which constitute the BBB. This localization in cerebral vessels of normal animals may constitute a possible yet poorly understood source of serum S100B upon disruption of the blood-brain barrier.

Our results are at variance with previous reports of S100B expression in non-CNS cells. Our results show restricted expression of mRNA for S100B while others have reported “expression” in many organs beyond testis, CNS. The term “expression” was often used to describe the presence of S100B *protein* while in this study we refer to expression only when mRNA was present. Data related to S100B distribution in tissues can be summarized as follows: 1) mRNA was reported in lung tissue from *C57BL6* mice [27] but our results were obtained in rats suggesting that species-specific differences may be involved; expression in cardiac myocytes was also reported, but again these were pathological samples or cells in culture [25], [26]; 2) folliculostellate cells in the anterior pituitary gland express mRNA for S100B when grown in culture [72]; these cells share many properties with brain astrocytes, including their capacity to transform into non-CNS cells; 3) Lymphocytes contain S100B protein, but there is no direct evidence that these cells also express the messenger for S100B. In fact, our results clearly show that the circulating S100B preferentially accumulates in immune cells. It is however possible that sub-populations of cells express mRNA for S100B and that this message was undetectable under our experimental conditions. It is also worth noting that cultured cells behave differently from their *in situ* counterparts and that transcription in vitro is regulated by many factors, including media, presence of a substrate, shear stress, etc.

The presence of S100B protein in dendritic cells is not unexpected given the findings by others [2], [73]. We did however describe the origin of this protein in lymphatic tissue. Since no mRNA for S100B was found in lymphatic organs or cells (Table 1), the most parsimonious explanation for S100B presence in quiescent lymph cells is cellular uptake. This was shown by our tracer experiments where a rapid (hours) uptake of S100B (and to a much lesser extent of S100A1) was measured. The content of S100B correlated with serum levels, as shown in the pilocarpine *status epilepticus* experiments. Thus, taken together our results have shown that lymph node and dendritic cell S100B derives from cellular uptake from a serum pool which in turn is controlled by blood-brain barrier disruption.

If S100B is segregated within barrier organs, why were measurable levels of S100B found in human [24] or rodent tissues (Table 1). Or, in other words, why are measurable serum levels present under normal steady-state conditions? Our experiments did not directly address this issue but several not mutually exclusive explanations may be formulated. For example, the BBB may not be a static membrane and occasional “leaks” occur to clear perhaps excessive levels of protein or metabolites. This is supported by several studies showing that under normal conditions BBB permeability is extremely low but amenable for small changes within physiological conditions [74]–[76]. The serum marker correlate shows that levels of S100B may vary within a normal range, which has been established for clinical tests to be ∼0.1 ng/ml [15], [19], [40]. But if normal conditions allow spillage of CNS protein in blood, why are then extravasations of S100B as seen during iatrogenic or seizure-related blood-brain barrier disruption leading to an immunogenic response? While current understanding of S100B pathophysiology is clearly incomplete, it has been shown that S100B acts on RAGE receptors only at extremely high, non-physiological concentrations (*e.g*., [27] where levels 100–50000x greater than serum levels were used). It is however possible that concentrations of ligand at immunological synapses may be greater that levels in body fluids. In addition, it is likely that low levels of S100B molecules induce a level of immune tolerance. Low levels of autoreactive B cells with self-antigens in the periphery ensure that highly autoreactive T cells are negatively selected and die to prevent overly self-reactive T cells from getting into the periphery. In addition, unlike the presentation of foreign antigens by mature dendritic cells, the presentation of self-antigens by immature dendritic cells neither activates nor matures the dendritic cells to express high levels of co-stimulatory molecules. However, the possibility that RAGE plays a role in the internalization process of S100B cannot be ruled out.

The significance of the presence of S100B in barrier organs is not well understood, nor has the role of S100B been entirely elucidated. However, the main scope and result of our work relate to the presence of S100B in immune cells. Again, little is known about the function of S100B but several reports have linked its presence to binding to RAGE receptors which are mediators of inflammation [77]. A novel finding of this study is that in addition to binding to extracellular domains of the RAGE receptors, S100B is also internalized by dendritic or other immune cells. This may constitute a step of self-antigen recognition, which is an initiator of the autoimmune response. Perhaps not surprisingly, when we analyzed blood from patients undergoing repeated BBBD for therapeutic purposes, we found that after a few months (or a few BBBD procedures) patients began to display autoantibodies against S100B. This suggests that antigen unmasking by BBB breakdown or leakage of S100B from endothelial cells may be the initiator of an autoimmune response.

Therapeutic or paroxysmal BBBD is a trigger for seizures ([9], [13], [50], [61], [63], [78] and Figure 6A). Our results present an unexpected consequence of cerebrovascular integrity failure: extravasated protein (S100B) was internalized in immune cells (Figure 5), and significant accumulation of IgGs was found in regions of BBBD in chronic epileptics [36]. We propose the following scenario. BBBD, regardless of its cause, results in extravasation of CNS antigen that is perceived as a danger associated pattern (DAMP). DAMPs are molecules that can initiate and perpetuate immune response in the noninfectious inflammatory response. S100B is internalized by specialized immune cells and the response remains in a quiescent state until a repeated BBBD event occurs. The presence of S100B is naïve tissue can be interpreted as a steady-state condition that is rekindled by a seizure or another event associated with BBBD such as an iatrogenic procedure. Repeated BBBD result in production of antibodies which may persist in blood without a significant consequence since the antigen is shielded by the BBB. When yet again BBB integrity fails, antibodies and autoantibodies enter the brain where they home in neurons and glia. This was demonstrated in two recent papers [36], [79]The significance of the latter may be pathologic as suggested by [80] or perhaps cytoprotective since in chronic epilepsy surviving neurons and glia containing IgGs were shown to have normal in morphology and nuclear condensation).

S100B autoantibodies have been described in a variety of diseases but primarily in Alzheimer's dementia [34], [35], [81]. In a study [31], it was shown that S100B autoantibodies appear in blood before antibodies against amyloid, rising earlier in disease course prior to peak levels of amyloid antibodies. More recently, the effects of BBBD triggered by repeated subconcussive episodes in football players included a post-game increase of S100B followed by IgG production against this marker and other CNS protein [37]. Levels of IgG s correlated with indices of altered MRI findings, suggesting a pathologic consequence of S100B autoimmunity. It does therefore appear that autoimmunity against brain proteins may be one of the initial steps in the progression towards post-traumatic cognitive decline. Several studies have underscored that S100B is not exclusively expressed in the brain [82]. These studies, however, measured primarily *protein* content of various cell types or organs. This widespread distribution of S100B *protein* beyond barrier organs appears to be due based on our data, to diffusion rather than expression and transcription. In order to account for possible artifacts or uncontrolled experimental variables, we used S100A1 as a tracer to compare to S100B. Since S100B and S100A1 have virtually identical molecular weights (Table 2), we were not surprised by the fact that they diffused freely and in a similar fashion. Our results have in fact shown that both proteins show some levels of uptake in immune cells and background diffusion in various organs. The latter is likely due to the fact that outside of barrier organs, vessels are “leaky” and therefore allow passage of low molecular weight protein. However, when quantifying results from immune cells, which were the most prominent cells displaying uptake of these proteins, we found that S100A1 was accumulated by these cells barely above background levels while the ingress of S100B was substantial

A limitation of our pilot human study in Figure 6 is that the patients who expressed autoantibodies against S100B were also affected by a brain tumor [50]. This may have caused a paraneoplastic reaction that may justify the presence of autoantibodies (*e.g*., [83]). However, the increases in S100B autoantibody titers were calculated as a percentage increases over baseline levels drawn prior to the initiation of therapy and long after the tumor was diagnosed. A sharp increase was observed only following repeated disruptions of the BBB as shown in Figure 6 and this phenomenon was only observed in patients where repeated spikes in serum S100B were measured.

In conclusion, our results show an unexpected role for S100B in the regulation of the neuroimmune response. Whether this response is mediated by RAGE receptors as suggested by others [84] remains unclear, but uptake of S100B was prominent in cells that are known to express receptors involved in immunomodulation. The presence of baseline levels of S100B in immune cells suggests a mechanism of immune tolerance for this CNS and CSF protein. Repeated serum S100B spikes as those occurring of BBBD, or as in a study by artificial elevation of levels by intravenous injection, may be sufficient to trigger a response that needs to be further elucidated in patients and experimental models. Our results also provide evidence that under pathological conditions such as seizures (Figure 5), the uptake of S100B by immune cells is greatly exaggerated. In fact, pilocarpine-treated animals exhibited elevated levels of S100B in spleen and in other immune organs. This suggests that the immune tolerance for the typically low levels of circulating S100B is overwritten by sudden or repeated increases in this protein. Repeated increases of S100B may thus become boosters of an autoimmune response against the brain, which may slowly but inexorably result in chronic neurological disease. This hypothesis is supported by the presence of elevated levels of IgG in the brain of epileptics (Figure 6). Repeated seizures are known to cause cognitive dysfunction and memory impairment. This may be one of the steps that produce pathological outcomes in chronic epilepsy as well as in diseases such as Alzheimer's dementia.

## Conclusions

S100B is only synthesized in barrier organs which are immunoprivileged. Our results suggest that this serum marker of BBBD may cause an autoimmune reaction by activation of immune cells that perceive this protein as a foreign antigen due to antigen unmasking.

## Supporting Information

Figure S1 **Raw data of representative mRNA results shown in Figure S1.** The panels show positive and negative experimental controls (A), positive (brain) and negative (polymorphonucleated cells) biological controls, as well a broad array of issue data.
(TIF)

Figure S2 **S100B levels in peripheral tissues are low at steady state and increase with rising serum S100B levels.** The rat hepatic tissue shown demonstrates a level of labeling with injected AlexaFlour 488-tagged S100B proportional to the amount of protein administered; use of a 1∶1 dilution (S100B to vehicle) produced more intense labeling (*L*) than that of a 1∶10 dilution (*R*). Note that the staining produced by use of a lower concentration of S100B resembled that of the images obtained by immunohistochemistry of most peripheral tissues, suggesting that the lack of signal in the latter images is due to low or negligible levels of S100B at steady state in non-CNS tissues. Satellite cells (*boxes*) label strongly with high circulating levels of S100B, but staining also persists, albeit to a lower degree, in tissues exposed to a lower concentration. IV injection consisted of AlexaFlour 488-tagged S100B at either a 1∶1 or 1∶10 dilution with phosphate-buffered saline (PBS) introduced via tail vein and allowed to circulate 2–3 hrs. prior to tissue harvesting.
(TIF)

Figure S3 **S100B uptake by CD86 positive dendritic cells in skin and spleen.** (**A**) Skin cells demonstrating S100B uptake are Langerhans dendritic cells expressing CD86. (**B**) Spleen cells (shown as CD4+ and CD8+ in Figure 5), are dendritic cells expressing the marker of dendritic cell stimulation CD86.
(TIF)

Figure S4 **Time- dependent uptake of S100B but not S100A1 by dendritic cells.** The cells were first extensively characterized for expression of CD86, CD11a and CD83, markers of mature dendritic cells. Robust uptake, albeit in less than 20% of cells was seen. These results are essentially the same as those reported in vivo, but cultured cells were easier to study since they constitute an ideal tool for time-dependent analysis.
(TIF)
